# Knowledge about type 2 diabetes: its impact for future management

**DOI:** 10.3389/fpubh.2024.1328001

**Published:** 2024-03-08

**Authors:** Pedro L. Ferreira, Carminda Morais, Rui Pimenta, Inês Ribeiro, Isabel Amorim, Sandra Maria Alves, Luiz Santiago

**Affiliations:** ^1^Centre for Health Studies and Research of the University of Coimbra, Portugal (CEISUC), Coimbra, Portugal; ^2^Faculty of Economics, University of Coimbra, Portugal (FEUC), Coimbra, Portugal; ^3^Superior School of Health of the Polytechnical Institute of Viana do Castelo (ESS-IPVC), Viana do Castelo, Portugal; ^4^School of Health, Polytechnic of Porto (ESS|P.PORTO), Porto, Portugal; ^5^Faculty of Medicine, University of Coimbra, Portugal (FMUC), Coimbra, Portugal

**Keywords:** diabetes, knowledge, quality of life, self-management, prevention

## Abstract

Diabetes can cause several long-term complications. Knowledge about this disease can play an important role in reducing diabetes-related complications. In addition, the lack of awareness leads to misconceptions, which joined with inadequate knowledge, are relevant barriers to proper diabetes management. In this study, we aimed to assess the diabetes knowledge of a type 2 diabetes (T2D) population and identify major knowledge gaps, in order to prevent complications and to increase quality of life. In a cross-sectional, observational study in a convenience sample, we identified individuals diagnosed with T2D attending ambulatory visits from five health settings, older than 18 years, with a time diagnosis of at least 1 year, and attending multidisciplinary visits for at least 3 months. To assess the knowledge of T2D individuals, we applied the Portuguese version of the Diabetes Knowledge Test. The sample included a total of 1,200 persons, of whom almost half were female. The age range of the participants varied from 24 to 94 years old, and the mean age was 65.6 ± 11.4 years. Most of the sample had a level of education under secondary and lived with someone. In our sample, 479 (39.9%) were insulin-treated. The percentage of correct answers was 51.8% for non-insulin vs. 58.7% for insulin treated (*p* < 0.05). There were three items with a percentage of correct answers lower than 15%; the item with the lower value of correct answers was the one related to the identification of signs of ketoacidosis with only 4.4% of correct answers, the errors presented a random pattern; the item related to the identification of which food should not be used to treat low blood glucose with 11.9%, where 56.9% of the sample’s participants considered that one cup of skim milk would be the correct answer (53.1% in non-insulin patients and 62.6% in insulin treated patients; *p* < 0.001). The item regarding the knowledge of free food presented a 13.3% of correct answers (10.8% non-insulin group vs. 17.1% insulin group; *p* < 0.01). Two of the three items with lower value of correct answers were related to glycemic control and health status monitoring, the other was related to diet and food.

## 1 Introduction

Diabetes is a chronic condition, which occurs when the body does not produce enough insulin or cannot use it in an effective away. Type 2 diabetes (T2D) is the most common form of this disease representing more than 90% of the all diabetes cases (1). It can be prevented or delayed by a healthy lifestyle and use of medication (2–4).

According to Diabetes Atlas 10th edition, there were 537 million individuals with diabetes in 2021. This number is expected to grow to 783 million by 2045 (1). The mean rate of people with Diabetes in the Organization for Economic Cooperation and Development (OECD) is 6.7%, and Portugal is above this value, with a rate of 9.8% (5).

Diabetes can cause several long-term complications such as lower limb amputation, cardiovascular diseases, retinopathy, neuropathies, and nephropathy (6, 7). Diabetes was responsible for 6.7 million deaths worldwide, in 2021. In Europe, one in three adults with diabetes is not diagnosed (1), increasing the probability of having a more severe disease and more diabetes-related complications.

There is no cure for diabetes and its management goes far beyond the medication. According to Hill (8), T2D leads to some adverse outcomes and is part of a cyclical process. This process includes socioeconomic determinants such as education, income and access to a healthy nutritional diet, lifestyle factors like dietary choices, physical activity and access to primary health care, and disease management (8). Genetics is ever more being involved in T2D etiology by predicting the risk of developing it (9), but it is the physical and social determinants which influence development and progression of the T2D (8). T2D conventional treatments are combined with strategies of behavioral changes, dietary improvements, physical activity, and treatment monitorization in order to properly manage this condition (10, 11).

People with diabetes need to monitor their health status, in particular by monitoring complications, care (12), and medication regimens (8). Therapeutic adherence is essential to achieve glycemic control and to avoid complications, together with physical activity, diet, and medication adjustment (13). People with this disease seem to comply with routine medication intake (14, 15), but compliance with lifestyle recommendations is less than desired (14–16). Chane-Po et al. (17) assessed the knowledge of a T2D population and revealed that the areas with the lowest scores were follow-up, diet, and physical activity. This highlights the need to inform the population, not only about the consequences of this disease, but also about the importance of adopting a healthy lifestyle and a proper disease management.

Knowledge about this disease can then play a significant role in reducing diabetes-related complications and improve its prevention (18, 19). Diabetes knowledge among people with this disease is associated to several sociodemographic factors, such as family history (19–22), educational status (20–22), family income (20), and exposure to health education (22). Therefore, Alemayehu et al. (23) determined the level of diabetes knowledge in a non-diabetes population and the results were not promising, 51.4% of the participants showing good knowledge about this disease, indicating the need of health education interventions.

Diabetes education can then be crucial to change people’s behaviors, promote disease self-management, and improve health outcomes (24, 25). A systematic review and a meta-analysis of randomized controlled trials highlighted the importance of educational interventions, by increasing the diabetes-related knowledge and improving glycated hemoglobin rates (26). To Kugbey et al. (27), illness perception and diabetes knowledge are also important determinants to diabetes self-care practices. In addition, diabetes knowledge affects the process of decision-making regarding physical exercise, diet, medication use, and health status monitoring which includes diabetes assessment and foot care (28).

The lack of awareness leads to misconceptions, which joined with inadequate knowledge are relevant barriers to a proper diabetes management (23, 29). Some studies showed, for instance, that 45% of people with diabetes think that “only sugar can affect blood sugar” (30), 47% believe that “bitter foods reduce the elevated blood sugar levels,” and 21.2% consider that diet does not play an important role in diabetes treatment (31). These studies also evidenced knowledge gaps about the best self-care practice (30), and regarding medication and its effects. For example, 36.5% believe that serious side effects can show up due to a prolonged use of oral hypoglycemic drugs and insulin, and 31% people with diabetes can eat anything they want if they take the medication. Additionally, 35.5% of the participants consider that lifestyle changes do not have a role in disease management (31).

Consequently, diabetes knowledge, self-management, and self-care practices are crucial factors with a potential significant impact in patients’ quality-of-life (32). Therefore, it is important to measure the knowledge people with diabetes have about their own disease. Such knowledge makes it possible to identify the topics or terms with which they are less aware of, and act accordingly. This allows the development of interventions to increase the knowledge of these patients, targeting the most critical areas, and helping them to prevent potential risk behaviors, or to be aware to serious signs of disease complications.

In this study, we aimed to assess the diabetes knowledge of a T2D population and to identify their major knowledge gaps, in order to prevent complications and to increase quality of life.

## 2 Methods

### 2.1 Study design

We carried out a descriptive and observational cross-sectional study in five health settings, one ambulatory department of a large hospital center, three health centers, and one diabetes specialized hospital. The research project was approved by the Ethical Committee of the Northern Regional Health Authority (Ref. 62/2018).

### 2.2 Sample

We identified individuals diagnosed with T2D attending ambulatory visits, older than 18 years, with a time from diagnosis at least 1 year and attending consultation in 3 months period in 2022. Patients diagnosed with cognitive impairment or mental illness were not included in this study. The participants completed the questionnaire independently, unless they were unable to read or write. In this case, a person helped to fill the questionnaire, by reading the questions with the minimum possible interpretation and without conditioning the answer.

All patients with diabetes who came for appointments at the five centers were approached sequentially. Data were collected while patients were waiting for the consultation to avoid possible appointment information bias.

### 2.3 Measurement instruments

To assess the knowledge of T2D individuals, we applied the Portuguese version of the Diabetes Knowledge Test (DKT). This is a self-administered instrument developed in the 90s by the Michigan Diabetes Research Training Center and it aims to measure the knowledge patients have about diabetes. It was developed for individuals with type 1 or type 2 diabetes, and it takes around 15 min to complete. This questionnaire is formed by two parts: the first one has 14 items, and it can be completed by anyone with diabetes; and the second part with nine items, and it was designed only for individuals with diabetes receiving insulin therapy. The final score is calculated according to the number of correct answers (33).

The DKT presents six dimensions, including: (i) food, with four items about the composition of food, its safety and which types of food should be avoid; (ii) ways to assess diabetes, with two items regarding methods to assess this disease; (iii) effect of external variables on diabetes control, with six items such as physical exercise or infection, on diabetes control; (iv) signs and symptoms, containing three items concerning the symptoms associated with natural evolution of diabetes and failures on its monitoring; (v) control over medication and its effects, with six items measuring individual’s response to adversities or forgetting to take insulin; and (vi) causes of glycemic deregulation, with two items aimed at understanding the individual’s perception of possible causes that may change blood glucose levels (34).

The reliability and the validity of the DKT were initially tested by Fitzgerald et al. (33). Internal consistency was good in both non-insulin and insulin subscales, with Cronbach’s alpha values greater than 0.70. To evaluate the construct validity, the authors formulated some hypotheses. These results showed that DKT scores were significantly higher in individuals with higher education levels, with type 1 diabetes and who attended an educational program regarding diabetes therapy (33). DKT showed good reliability in Portuguese population with Cronbach’s alpha higher than 0.8 and a positive correlation with disease control (34). In Portugal, this measurement instrument has been used to assess the association between knowledge about diabetes and the ability to control this disease (35, 36).

In this study we also included sociodemographic (sex, age, education, and living arrangements) and clinical variables [body mass index (BMI), glycated hemoglobin (HbA1c), treatment with insulin or not, and time of diabetes diagnosis] of the participants. Clinical-related and diagnostics data were collected from the clinical files. Physical exercise was self-reported and it was considered as positive if the patient reported more than 1 h per week.

### 2.4 Statistical analysis

Initially, data were analyzed using descriptive statistics to characterize the sample, observe the distribution of the wrong and correct answers between non-insulin and insulin-treated groups, and to understand the participant’s choices among response options. To present these data, we grouped DKT answers in four major groups that may determine the diabetes-related knowledge people have with their disease: (i) diet and food; (ii) physical activity; (iii) glycemic control and health status monitoring; and (iv) medication and treatment.

Inferential statistics was also conducted, including the binomial test and chi-square test of independence. The binomial test was performed to compare percentages between insulin and non-insulin treated diabetic patients. The chi-square test of independence was used to understand whether there was any relationship between non-insulin/insulin-treated groups and the distribution of answers.

Data were analyzed using IBM SPSS Statistical Package for the Social Sciences version 28 and missing data were handled using the pairwise approach.

## 3 Results

### 3.1 Sample characteristics

There were no refusals. The sample included a total of 1,200 persons, of whom almost half were female. The age range of the participants varied from 24 to 94 years old, and the mean age was 65.6 ± 11.4 years. Most of the sample had a level of education under secondary and lived with someone. There were no statistically significant differences between insulin and non-insulin treated patients concerning sex (χ^2^ = 0.117; *p* = 0.733) and educational level (χ^2^ = 0.076; *p* = 0.782). Naturally, individuals over the age of 65 are more likely to be treated with insulin (χ^2^ = 21.937; *p* < 0.001). The information about sociodemographic and clinical variables of the sample is presented in Table 1.

**Table 1:** Sociodemographic and clinical characteristics of the observed sample (*n* = 1,200).

Variable	Value	*n*	%
Setting of care	Health center	530	44.2
	Hospital ambulatory	260	27.1
	Diabetes specialized hospital	410	34.2
Sex	Male	599	49.9
	Female	601	50.1
Age (years)	< 65	486	40.5
	≥ 65	714	59.5
	Min–Max	24–94	
	Q1–Q3	58–74	
	Mean ± standard deviation	65.6 ± 11.4	
Education	Under secondary	1,008	84.3
	Secondary or above	188	15.7
Living arrangement	Living alone	175	14.7
	With company	1,015	85.3
Time after diagnosis (years)	≤ 3	196	25.1
	>3	584	74.9
	Min–Max	0–50	
	Q1–Q3	3–15	
	Mean ± standard deviation	10.7 ± 9.2	
HbA1c (%)	<6.5	313	32.4
	[6.5–8]	445	46.1
	>8	207	21.5
	Min-Max	4.9–13.6	
	Q1–Q3	6.2–7.9	
	Mean ± standard deviation	7.2 ± 1.3	
BMI (Kg/m^2^)	Underweight (< 18.5)	3	0.3
	Normal weight (18.5–24.9)	186	15.8
	Overweight (25–29.9)	524	44.6
	Obesity (≥30)	462	39.3
	Min–Max	18.4–66.3	
	Q1–Q3	26.2–32.0	
		
	Mean ± standard deviation	29.5 ± 5.1	
Insulin treatment	Yes	479	39.9
	No	721	60.1
Complications	Yes	257	39.4
	No	396	60.6
Follow diet	Yes	456	58.2
	Sometimes	248	31.7
	No	79	10.1
Physical exercise	Yes	473	72.4
	No	180	27.6

BMI, Body mass index; HbA1c, Glycated hemoglobin; Min, Minimum; Max, Maximum; and Q1, Q3, First and third quartiles.

In our sample, 479 (39.9%) were insulin-treated. The other 721 (60.1%) participants were on special diet with/without oral antidiabetics. Most of these patients (74.9%) had been diagnosed at least 3 years ago, almost half (46.1%) of them showed a glycated hemoglobin between 6.5 and 8.0%, and 83.9% were overweighed or obese. Almost 40% presented complications, mainly coronary heart disease (17.2%) and retinopathy (15.1%). Only 10.1% do not follow a special diet and the majority (72.4) physical exercise.

Based on the four analytic groups previously defined the implementation of DKT revealed.

### 3.2 Diet and food

The descriptive statistics of correct answers regarding diet and food questions of the DKT are presented in Table 2.

**Table 2:** Descriptive statistics of correct answers for the DKT regarding diet and food.

Item	All sample	Non-insulin treated	Insulin-treated
	*n* (%)	*n* (%)	*n* (%)
DKT-01: The diabetes diet is			
− The way most people eat	115 (9.6)	66 (9.2)	49 (10.2)
− Healthy diet for most people^†^	856 (71.3)	503 (69.8)	353 (73.7)
− Too high in carbohydrate for most people	50 (4.2)	28 (3.9)	22 (4.6)
− Too high in protein for most people	130 (10.8)	83 (11.5)	47 (9.8)
− Missing data	49 (4.1)	41 (5.7)	8 (1.7)
DKT-02: Which of the following is highest in carbohydrate?			
− Baked chicken	186 (15.5)	105 (14.6)	81 (16.9)
− Swiss cheese	145 (12.1)	73 (10.1)	72 (15.0)
− Baked potato^†^	678 (56.5)	412 (57.1)	266 (55.5)
− Peanut butter	122 (10.2)	75 (10.4)	47 (9.8)
− Missing data	69 (5.8)	56 (7.8)	13 (2.7)
DKT-03: Which of the following is the highest in fat?			
− Low fat milk^†^	470 (39.2)	285 (39.5)	185 (38.6)
− Orange juice	55 (4.6)	32 (4.4)	23 (4.8)
− Corn	237 (19.8)	129 (17.9)	108 (22.5)
− Honey	360 (30.0)	215 (29.8)	145 (30.3)
− Missing data	78 (6.5)	60 (8.3)	18 (3.8)
DKT-04: Which of the following is a “free food”?			
− Any unsweetened food	518 (43.2)	311(43.1)	207 (43.2)
− Any diabetic food	327 (27.3)	207 (28.7)	120 (25.1)
− Any food that says “sugar free” on the label	144 (12.0)	84 (11.7)	60 (12.5)
− Any food that has less than 20 calories per serving^†^	160 (13.3)	78 (10.8)	82 (17.1)
− Missing data	51 (4.3)	41 (5.7)	10 (2.1)

^†^Correct answer.

In the first two items (DKT-01 and DKT-02), the percentage of correct answers was significantly higher than to flip a coin, respectively, 71.3 and 56.5%. We should highlight the percentage of error greater than 80% in the identification of “free food” in any of the groups.

Comparing the wrong answers among non-insulin and insulin treated patients, a significant higher percentage (10.1 vs. 15.0%; *p* = 0.019) of insulin-treated patients mentioning that Swiss cheese is the highest in carbohydrate. We also should note that patients treated with insulin more often responded correctly to what may be classified as “free food” (10.8 vs. 17.1%; *p* < 0.01).

### 3.3 Physical activity

Table 3 shows the results of the only DKT item addressing physical activity. Recalling that 72.4% of the participants in this study practice physical activity, it is not surprising that 83.3% of the whole sample gave a correct answer, demonstrating good knowledge of the influence of physical activity on disease management. However, some non-insulin treated (11.1%) and insulin treated patients (6.3%) still maintain the belief that physical exercise has no effect on blood glucose.

**Table 3:** Descriptive statistics of answers for the DKT regarding physical activity.

Item	All sample	Non-insulin treated	Insulin-treated
	*n* (%)	*n* (%)	*n* (%)
DKT-09: For a person in good control, what effect does exercise have on blood glucose?			
Lowers it^†^	999 (83.3)	577 (80.0)	422 (88.1)
Raises it	60 (5.0)	37 (5.1)	23 (4.8)
Has no effect	110 (9.2)	80 (11.1)	30 (6.3)
Missing data	31 (2.6)	27 (3.7)	4 (0.8)

^†^Correct answer.

### 3.4 Glycemic control and health status monitoring

This group includes DKT items presented in Table 4, regarding ways to assess diabetes, the effect of external variables, signs and symptoms to look out for, and causes of glycemic deregulation.

**Table 4:** Descriptive statistics of answers for the DKT regarding glycemic control and health status monitoring.

Item	All sample	Non-Insulin Treated	Insulin-Treated
	*n* (%)	*n* (%)	*n* (%)
DKT-05: Glycosylated hemoglobin (hemoglobin A1) is a test that is a measure of your average blood glucose level for the past:			
Day	103 (8.6)	60 (8.3)	43 (9.0)
Week	100 (8.3)	61 (8.5)	39 (8.1)
6–10 weeks^†^	383 (31.9)	201 (27.9)	182 (38.0)
6 months	386 (32.2)	267 (37.0)	119 (24.8)
Missing data	228 (19.0)	132 (18.3)	96 (20.0)
DKT-06: Which is the best method for testing blood glucose?			
Urine testing	70 (5.8)	35 (4.9)	35 (7.3)
Blood testing^†^	927 (77.3)	548 (76.0)	379 (79.1)
Both are equally good	173 (14.4)	111 (15.4)	62 (12.9)
Missing data	30 (2.5)	27 (3.7)	3 (0.6)
DKT-07: What effect does unsweetened fruit juice have on blood glucose?			
Lowers it	100 (8.3)	63 (8.7)	37 (7.7)
Raises it^†^	768 (64.0)	428 (59.4)	340 (71.0)
Has no effect	291 (24.3)	194 (26.9)	97 (20.3)
missing data	41 (3.4)	36 (5.0)	5 (1.0)
DKT-08: Which should not be used to treat low blood glucose?			
3 hard candies	210 (17.5)	133 (18.4)	77 (16.1)
½ cup orange juice	94 (7.8)	57 (7.9)	37 (7.7)
1 cup diet soft drink^†^	143 (11.9)	92 (12.8)	51 (10.6)
1 cup skim milk	683 (56.9)	383 (53.1)	300 (62.6)
Missing data	70 (5.8)	56 (7.8)	14 (2.9)
DKT-10: Infection is likely to cause			
An increase in blood glucose^†^	805 (67.1)	439 (60.9)	366 (76.4)
A decrease in blood glucose	71 (5.9)	46 (6.4)	25 (5.2)
No change in blood glucose	196 (16.3)	135 (18.7)	61 (12.7)
Missing data	128 (10.7)	101 (14.0)	27 (5.6)
DKT-11: The best way to take care of your feet is to:			
Look at and wash them each day^†^	999 (83.3)	577 (80.0)	422 (88.1)
Massage them with alcohol each day	19 (1.6)	12 (1.7)	7 (1.5)
Soak them for one hour each day	61 (5.1)	45 (6.2)	16 (3.3)
Buy shoes a size larger than usual	111 (9.3)	77 (10.7)	34 (7.1)
Missing data	10 (0.8)	10 (1.4)	0 (0)
DKT-12: Eating food lower in fat decreases your risk for			
Nerve disease	62 (5.2)	39 (5.4)	23 (4.8)
Kidney disease	131 (10.9)	76 (10.5)	55 (11.5)
Heart disease^†^	872 (72.7)	511 (70.9)	361 (75.4)
Eye disease	59 (4.9)	34 (4.7)	25 (5.2)
Nerve disease	76 (6.3)	61 (8.5)	15 (3.1)
DKT-13: Numbness and tingling may be symptoms of:			
Nerve disease^†^	572 (47.7)	372 (51.6)	200 (41.8)
Kidney disease	281 (23.4)	128 (17.8)	153 (31.9)
Eye disease	38 (3.2)	25 (3.5)	13 (2.7)
Liver disease	167 (13.9)	97 (13.5)	70 (14.6)
Missing data	142 (11.8)	99 (13.7)	43 (9.0)
DKT-14: Which of the following is usually not associated with diabetes?			
Vision problems	85 (7.1)	68 (9.4)	17 (3.5)
Kidney problems	53 (4.4)	38 (5.3)	15 (3.1)
Nerve problems	145 (12.1)	97 (13.5)	48 (10.0)
Lung problems^†^	839 (69.9)	448 (62.1)	391 (81.6)
Missing data	78 (6.5)	70 (9.7)	8 (1.7)
DKT-15: Signs of ketoacidosis include:			
Shakiness			91 (19.0)
Sweating			116 (24.2)
Vomiting^†^			21 (4.4)
Low blood glucose			108 (22.5)
Missing data			143 (29.9)
DKT-22: High blood glucose may be caused by:			
Not enough insulin^†^			295 (61.6)
Skipping meals			67 (14.0)
Delaying your snack			74 (15.4)
Large ketones in your urine			23 (4.8)
Missing data			20 (4.2)
DKT-23: Which one of the following will most likely cause an insulin reaction?			
Heavy exercise^†^			371 (77.5)
Infection			26 (5.4)
Overeating			23 (4.8)
Not taking your insulin			54 (11.3)
Missing data			5 (1.0)

^†^Correct answer.

In what concerns the two first items regarding ways to assess diabetes, only about one third of the participants (31.9%) gave a correct answer to the item about how much of the time is associated to the measure of hemoglobin A1 (DKT-05). There were 27.9% correct answers among the non-insulin treated patients and bit more (38.0%) among the insulin treated ones (*p* < 0.001).

On the contrary, in item about which is the best method for testing blood glucose (DKT-06), a significant consistency of the answers and high percentages of correct answers among the non-insulin treated patients (76.0%) as well as in the insulin treated patients (79.1%).

The effect of external variables on diabetes control was also asked, with significant statistical higher percentages of correct responses on all items (*p* < 0.001), the exception of the item about which should not be used to treat low blood glucose (DKT-08), for which a weak percentage of correct responses, less than 12%; *p* < 0.001, was found. In addition, 56.9% of the sample’s participants considered that one cup of skim milk would be the correct answer (53.1% in non-insulin patients and 62.6% in insulin treated patients; *p* < 0.001). An exception was the question on what happens when a diabetic patient eats food lower in fat (DKT-12), 72.7% of correct answers being revealed (70.9% in non-insulin treated and 75.4% in insulin patients; *p* = 0.089).

Comparing non-insulin and insulin treated patients, the latter ones showed higher levels of knowledge, significantly mentioning less frequently that unsweetened fruit juice had no effect on blood glucose (DKT-07; *p* = 0.011), as well as an infection (DKT-10; *p* < 0.01). For feet care (DKT-11), again, insulin treated patients showed smaller percent of wrong answers (*p* = 0.007).

For signs and symptoms, in all items, there were significant differences in the percentages of correct answers (*p* < 0,001), its behavior of differences not always being the same. When compared with non-insulin treated patients, insulin treated ones showed less knowledge for identification of numbness and tingling (DKT-13), where the most frequent error was the association of kidney disease, particularly in the group of patients treated with insulin. Insulin treated patients showed more knowledge about which symptoms are not usually associated with diabetes (DKT-14). Regarding signs of ketoacidosis (DKT-15), 4.4% of insulin treated patients gave a correct answer.

For glycemic control and health status monitoring, group of analysis addressing what may be caused by high blood glucose (DKT-22) and by an insulin reaction (DKT-23) were studied. In both items, only answered by insulin treated patients, we achieved higher percentages of correct answers, respectively (61.6 and 77.5%).

### 3.5 Medication/treatment

This group includes DKT questions presented in Table 5, addressing control over medication and its effects.

**Table 5:** Descriptive statistics of answers for the DKT regarding treatment.

Item	Insulin-treated
	*n* (%)
DKT-16: If you are sick with the flu which of the following changes should you make	
Take less insulin	16 (3.3)
Drink less liquids	22 (4.6)
Eat more proteins	72 (15.0)
Test for glucose and ketones more often^†^	348 (72.7)
Missing data	21 (4.4)
DKT-17: If you have taken intermediate-acting insulin (NPH or Lente), you are most likely to have an insulin reaction in:	
1–3 h	48 (10.0)
6–12 h^†^	190 (39.7)
12–15 h	97 (20.3)
More than 15 h	65 (13.6)
Missing data	79 (16.5)
DKT-18: You realize just before lunchtime that you forgot to take your insulin before breakfast. What should you do now?	
Skip lunch to lower your blood glucose	8 (1.7)
Take the insulin that you usually take before breakfast	119 (24.8)
Take twice as much insulin as you usually take at breakfast	7 (1.5)
Check your blood glucose level to decide how much insulin to take^†^	300 (62.6)
Missing data	45 (9.4)
DKT-19: If you are beginning to have an insulin reaction, you should:	
Exercise	15 (3.1)
Lie down and rest	73 (15.2)
Drink some juice^†^	328 (68.5)
Take regular insulin	26 (5.4)
Missing data	37 (7.7)
DKT-20: Low blood glucose may be caused by:	
Too much insulin^†^	309 (64.5)
Too little insulin	61 (12.7)
Too much food	32 (6.7)
Too little exercise	55 (11.5)
Missing data	22 (4.6)
DKT-21: If you take your morning insulin but skip breakfast your blood glucose level will usually	
Increase	68 (14.2)
Decrease^†^	350 (73.1)
Remain the same	24 (5.0)
Missing data	20 (4.2)

^†^Correct answer.

The distribution of all items is not at random and the percentage of correct responses is mostly higher than 60%, with the exception of the item that addresses the probability of having an insulin reaction when taking intermediate-acting insulin NPH or Lente (DKT-17). In this item, the error distribution has an incidence of about 39.7% in the 6–12 h response.

For all other items, Table 5, more than 60% of insulin treated patients responders gave the right answer about changes needed to be make when they are sick with flu (DKT-16), what to do if forgot to take insulin before breakfast (DKT-18) and what may happen to the blood glucose level in this case (DKT-21), or when having an insulin reaction (DKT-19) and about the expected causes of low blood glucose (DKT-20).

### 3.6 Determinants of the level of knowledge

As determinants of the level of knowledge of people with diabetes, whether treated with inulin or not, Tables 6, 7 present the results of the tests carried out.

**Table 6:** Non-insulin treated patients.

Variable	Value	*n*	M ± SD	|*t*| or *F*	*Sig*
Setting of care	Health center	448	0.55 ± 0.18	24.465	<0.001
	Hospital ambulatory	76	0.65 ± 0.12		
	Diabetes specialized hospital	197	0.49 ± 0.13		
Sex	Male	357	0.54 ± 0.17	0.851	0.395
	Female	364	0.55 ± 0.17		
Age (years)	< 65	253	0.55 ± 0.16	3.817	<0.001
	≥ 65	468	0.52 ± 0.17		
Education	Under secondary	606	0.52 ± 0.16	6.829	<0.001
	Secondary or above	111	0.64 ± 0.16		
Living arrangement	Living alone	103	0.50 ± 0.17	2.327	0.020
	With company	609	0.55 ± 0.17		
Time after diagnosis (years)	≤ 3	163	0.51 ± 0.19	4.699	<0.001
	>3	351	0.59 ± 0.16		
HbA1c (%)	<6.5	259	0.55 ± 0.18	1.544	0.123
	≥6.5	265	0.57 ± 0.17		
BMI (Kg/m^2^)	Underweight/Normal weight	56	0.55 ± 0.20	0.382	0.702
	Overweight/	352	0.57 ± 0.17		
Complications	Yes	125	0.54 ± 0.19	2.109	0.036
	No	283	0.58 ± 0.16		
Follow diet	Yes	335	0.56 ± 0.18	5.548	0.004
	Sometimes	128	0.58 ± 0.17		
	No	54	0.49 ± 0.17		
Physical exercise	Yes	329	0.56 ± 0.18	0.490	0.666
	No	79	0.57 ± 0.15		

BMI, Body mass index; HbA1c, Glycated hemoglobin; Min, Minimum; Max, Maximum; and Q1, Q3, First and third quartiles.

**Table 7:** Insulin treated patients.

Variable	Value	*n*	M ± SD	|*t*| or *F*	*Sig*
Setting of care	Health center	82	0.61 ± 0.18	46.715	<0.001
	Hospital ambulatory	184	0.67 ± 0.11		
	Diabetes specialized hospital	213	0.54 ± 0.14		
Sex	Male	242	0.60 ± 0.14	0.012	0.990
	Female	237	0.60 ± 0.15		
Age (years)	< 65	233	0.62 ± 0.15	2.899	<0.001
	≥ 65	246	0.58 ± 0.14		
Education	Under secondary	402	0.58 ± 0.14	5.560	<0.001
	Secondary or above	77	0.68 ± 0.12		
Living arrangement	Living alone	72	0.57 ± 0.14	1.947	0.05
	With company	406	0.61 ± 0.15		
Time after diagnosis (years)	≤ 3	33	0.63 ± 0.16	0.707	0.480
	>3	233	0.65 ± 0.13		
HbA1c (%)	<6.5	116	0.66 ± 0.12	1.947	0.135
	≥6.5	150	0.64 ± 0.14		
BMI (Kg/m^2^)	Underweight/ Normal weight	35	0.67 ± 0.15	0.570	0.369
	Overweight /	210	0.66 ± 0.13		
Complications	Yes	132	0.65 ± 0.12	1.373	0.171
	No	113	0.67 ± 0.14		
Follow diet	Yes	121	0.65 ± 0.15	4.503	0.012
	Sometimes	120	0.66 ± 0.11		
	No	25	0.57 ± 0.14		
Physical exercise	Yes	144	0.65 ± 0.14	0.622	0.535
	No	101	0.66 ± 0.11		

BMI, Body mass index; HbA1c, Glycated hemoglobin; Min, Minimum; Max, Maximum; and Q1, Q3, First and third quartiles.

Looking at the determinants of the level of knowledge, in general, as we can observe from these two last tables that the time after diagnosis and the absence of complications seem to be determinants of knowledge for patients not treated with insulin, the same not happening with those treated with insulin. Moreover, regardless of the use of insulin, being followed up in a hospital outpatient clinic, being under 65 years of age, having a higher education, living accompanied and following a diet proved to be determinants of the level of knowledge.

## 4 Discussion

Diabetes is a complex management disease whose effectiveness depends, to a large extent, on the ability of people to cope with their knowledge, will, skills, and treating team for control and management of the clinical situation (37–39). Thus, the control of this health condition, as well as the prevention and delay of complications, are directly linked to decisions made by people with T2D, namely regarding diet, physical exercise, monitoring of glycemic control, and medication (37).

Looking at variables sex, HbA1c (cut-off point: 6.5%), and BMI (cut-off point: 25 kg/m2), no determinants of knowledge were found between the two sub-samples of patients. That is, whether insulin-treated or non-insulin-treated patients, it is not the values of these variables that contribute more or less to the knowledge of people with diabetes. The same is true for physical exercise.

Regarding the setting of care, higher knowledge (*p* < 0.001) was found among patients followed in hospital outpatient clinics, although the worst level of knowledge was found among patients followed in the specialized diabetes hospital. Higher knowledge was also always found among patients who were younger (*p* < 0.001), had higher education (*p* < 0.001), did not live alone (*p* < 0.001), or followed a diet, even if not constantly.

The behavior of the above variables did not differ between the two subsamples of diabetic patients. However, in non-insulin-treated patients, time since diagnosis appears to be a determinant of knowledge about the disease for those with more than 3 years since diagnosis are more likely to have greater knowledge. Finally, there appears to be relative success in prevention, as those without complications are the most knowledgeable.

However, for appropriate decisions to be made, and for their own benefit, people with diabetes need to understand the nature of their condition and acquire the ability to control and manage it effectively. Knowledge is, thus, fundamental to an effective diabetes management (40), as it is one of the determinants for a person to become an effective partner in the care process (41) and therefore improve the quality of life (42). In fact, the prevalence of the Metabolic Syndrome (43) in which T2D is paramount is important in Portugal (44), where the T2D profile is one of lack of physical exercise, psychological distress, and inadequate feeding.

The current study aimed to contribute to an effective optimization of the margin of responsibility of health professionals, through the knowledge of the characteristics of the population, as well as their level of specific knowledge in the area, by identifying the main gaps to be considered in therapeutic education processes. The sociodemographic and clinical profile of the sample is similar to the findings of other national and international studies (41). Thus, most respondents are over 65 years old and have a low level of education (40, 45), which may justify the low level of knowledge, as well as the potential difficulty in understanding some items. From a clinical point of view, some of the results also point in the same direction: the need to change clinical practices. In this context, we highlight the high level of HbA1C (more than 20% higher than 8%), as well as overweight/obesity (present in more than 80% of respondents), thus showing a high risk of complications (46), in a pathology that, by itself, is silent (47).

In general, similar to other studies (34, 48–51), the level of knowledge of the respondents is low, given the high percentage of wrong answers (and non-answers) to the items asked in the six dimensions of the DKT. So what factors are there to explain this lack of knowledge: Bad treating team-T2D patient communication? Social health determinants?

In our study, the majority of the sample believes—and rightly so—that the ideal diet for diabetes is the healthy diet for most people, although about a quarter of the population does not have this same opinion. However, when asked about the different nutrients that make up food, a high percentage of incorrect answers was evident. For example, when asked about the richest foods in fats, 54% answered incorrectly, with 30% referring to “honey” or even 43.2% refer to any food without sugar. According to some authors (51), this finding may be related to the lower emphasis given to other nutrients (lipids and proteins), namely by health professionals, in health education sessions.

Diet is an integral part of virtually all therapeutic education processes with people with diabetes and their families (37, 52). A large variety of nutritional guidelines is available (47, 53, 54). Assuming that the diet should be diversified (47) and that most of the food eaten is transformed into glucose, there is a need to ensure conditions that guarantee conscious decision-making by people with diabetes. People with diabetes and their families should be equipped with the knowledge to understand the caloric value of foods, for example, from food labels, and to distinguish, in an appropriate way, which foods to use according to the balance that must be ensured between physical activity, the intercurrences they face in their daily lives, the imminent therapeutic (dis)controls (55), and the pleasure of eating (47).

Regular physical activity and dietary management are essential in non-pharmacological treatment, not only in diabetes, but also in the management of other pathologies and risk factors such as overweight, glycemic (dis)control (56) and cardiovascular diseases (37). These topics are part of the approaches of most health professionals and seem to be well known by the majority of the participants.

Although there is an acceptable level of knowledge regarding diet and exercise management, there is a need for further intervention to promote effective treatment of the disease (57). The health outcomes can be achieved by encouraging participation in educational programs, using individual and group strategies, motivational interviewing, and the involvement of peers in a logic of (co)creation (58). The aims of educational programs include providing knowledge and skills, and also changing the patients’ behavior, increasing their motivation to comply with therapeutic recommendations, in order to establish a partnership in the treatment process and prepare the patient for self-care (59).

Blood glucose monitoring is another key pillar of disease self-management, in order to avoid blood glucose “spikes,” commonly associated with acute and chronic complications (13, 60). In this dimension too, the knowledge gap is evident. If, on the one hand, the majority of respondents identify the blood test as the best method for testing blood glucose, on the other hand, when asked about HbA1c, the difficulties are notorious, particularly in the group of insulin-treated people where about 62% did not answer this question. This result is particularly serious, since the use of HbA1c is a recommended indicator not only as a diagnostic and clinical activity (61) but also for disease self-management and, consequently, for the prevention of complications (13). The high percentage of responses referring to 6 months as the period of time for the assessment of the average blood glucose level, i.e., HbA1c, may be explained by the at least biannual nature of the standards and guidelines issued by the Directorate-General for Health (61). This may be due to the fact that most people with diabetes may be unaware that HbA1c is an indicator of average blood glucose over the last 8–12 weeks, given that the average lifespan of red blood cells is 120 days (61, 62). Therefore, the potential for this knowledge to be effectively mobilized for disease self-management may be underutilized.

In general, it seems that insulin-treated participants have more T2D knowledge, particularly about the symptoms and complications, which may lead to a better self-care. According to Afaya et al. (37), who found similar results, this can be due to a higher contact with health professionals, or these could pay a special attention to insulin-treated patients in terms of education, to enable them to achieve better glycemic control, and so increasing their T2D knowledge. A specific access to healthcare for people with diabetes could improve knowledge, disease management, and health outcomes (49).

Considering only insulin-treated participants, the results revealed some fragilities, especially in the areas of insulin control and its effects, with 60.4% of the participants not answering correctly about the duration of action of the different types of insulin, 37.4% did not know what to do if they forget to take insulin, a similar percentage of participants were not aware about the possible reactions to insulin, and 27.3% did not know about the necessary changes to adopt in case of having a flu. This highlights the importance of promoting patient autonomy, paying special attention to communication and systematically informing patients about the most common medication errors among patients in order to improve safety.

The short contact time with health services, in Portugal and other countries (63, 64), and the fact that effective 2TD management involves training and empowerment, requires new forms of care (24, 42).

Therefore, in addition to monitoring knowledge, the ability of self-manage should be assessed, as well as support needs and lifestyle (65). It is also suggested that written information regarding the individual care plans, agreed with the healthcare team, should be provided.

However, this reorientation of care practice centered on people with diabetes and their families requires education and training for healthcare teams, specifically in the field of self-management (66–68).

## 5 Conclusion

Managing T2D continues to be a huge challenge for both health professionals and people with this disease. Knowledge is essential for an effective control of the disease, as it allows the patient to play an active and cooperative role in planning and monitoring the therapeutic, which promote informed decisions.

The use of this instrument allowed us to measure the knowledge that patients with T2D have about their disease, as well as to compare insulin-treated and non-insulin-treated patients. Although, in general, insulin-treated patients presented more knowledge about some topics of the questionnaire, we found that there were questions with relatively low percentages in both groups.

The results of this study reinforce the need to improve the disease knowledge of T2D patients and to enable them to achieve better health outcomes.

Some of the factors associated with the limited diabetes knowledge are modifiable, and can be addressed through more targeted interventions, associated with raising awareness of the importance of accessing services, particularly at community level. Researchers, health educators, and health professionals should be aware of the main areas that are less known by patients, so they can focus on these topics and carry out personalized interventions.

A therapeutic education plan designed together with the person with diabetes is crucial, so that they can understand the nature of the disease and also have the knowledge and skills to manage its symptoms and glycemic control. Therefore, disease self-management is very important and it can be the key to achieve sustainable behavior change and empower with the skills to successfully manage the disease. Educational interventions will always be complex and challenging, as well as the management of T2D itself and the evaluation of its effectiveness.

## Data availability statement

The raw data supporting the conclusions of this article will be made available by the authors, without undue reservation.

## Ethics statement

The studies involving humans were approved by Ethical Committee of the Northern Regional Health Authority, Ministry of Health. The studies were conducted in accordance with the local legislation and institutional requirements. The participants provided their written informed consent to participate in this study.

## Author contributions

PF: Conceptualization, Formal analysis, Methodology, Writing – original draft, Writing – review & editing. CM: Conceptualization, Methodology, Writing – original draft, Writing – review & editing. RP: Conceptualization, Formal analysis, Methodology, Writing – original draft, Writing – review & editing. IR: Writing – original draft, Writing – review & editing. IA: Writing – original draft, Writing – review & editing. SA: Formal analysis, Writing – original draft, Writing – review & editing. LS: Writing – original draft, Writing – review & editing.
